# Effect of Organic and Conventional Management on Bio-Functional Quality of Thirteen Plum Cultivars (*Prunus salicina* Lindl.)

**DOI:** 10.1371/journal.pone.0136596

**Published:** 2015-08-27

**Authors:** Francisco Julián Cuevas, Inmaculada Pradas, María José Ruiz‐Moreno, Francisco Teodoro Arroyo, Luis Felipe Perez-Romero, José Carlos Montenegro, José Manuel Moreno‐Rojas

**Affiliations:** 1 Postharvest Technology and Agrifood Industry Area, Andalusian Institute of Agricultural and Fishering Research and Training (IFAPA), Alameda del Obispo, Córdoba, Spain; 2 Organic Production and Natural Resources, Andalusian Institute of Agricultural and Fishering Research and Training (IFAPA), Las Torres-Tomejil, Seville, Spain; National Research Council of Italy, ITALY

## Abstract

In this study, thirteen Japanese plum cultivars (*Prunus salicina* Lindl.) grown under conventional and organic conditions were compared to evaluate the influence of the culture system on bioactive compounds. Their organic acids content (malic, citric, tartaric, succinic, shikimic, ascorbic and fumaric acid), total polyphenols, total anthocyanins, total carotenoids and antioxidant capacity (FRAP, ABTS) were evaluated. The study was performed during two consecutive seasons (2012 and 2013) in two experimental orchards located at the IFAPA centre Las Torres-Tomejil (Seville, SW Spain). The culture system affected all the studied parameters except for total carotenoid content. The organic plums had significantly higher polyphenol and anthocyanin concentrations and a greater antioxidant capacity. Additionally, significant differences between cultivars were also found. ‘Showtime’ and ‘Friar’ were the cultivars with the highest polyphenol concentration and antioxidant capacity. ‘Black Amber’ had the highest anthocyanin content and ‘Larry Ann’ and ‘Songold’ the highest carotenoid content. ‘Sapphire’ and ‘Black amber’ were the cultivars with the highest concentration of ascorbic acid. Our results showed a strong year effect. In conclusion, organic management had an impact on the production of phytochemical compounds in plums.

## Introduction

The genus *Prunus* includes an important number of fruit crops: peach (*P*. *persica*), apricot (*P*. *armeniaca*), cherry (*P*. *avium*), almond (*P*. *dulcis*) and plum (*P*. *salicina*). Plums are mainly consumed fresh and are becoming increasingly popular as a result of their attractive appearance and extraordinary flavour. Spain produces about 8% of the total European plum production (3^rd^ position) and is the main exporter (seasonal market) in the world [1].

Europeans demand high quality fresh products, especially those with high organoleptic quality and nutritional value [2]. For this reason, those characteristics have been important targets for breeding during the last decade [3]. A recent survey showed that the demand for organic products by European consumers increased fourfold during the last decade while the area devoted to their cultivation only doubled [4]. In Andalusia, a huge increase in the land dedicated to organic farming took place (sevenfold in the period, 2001–2013). At present, Andalusia produces more than 50% of the total Spanish production of organic fruit and vegetables [5].

European Union guidelines regarding organic production [6] forbids the use of synthetic products (fertilisers and plant protection methods). The principles for organic agriculture are similar in the different European countries and the inputs permitted are regulated by law.

On the one hand, several reviews and articles generally state that organic fruits and vegetables have a higher amount of micronutrients and health-related secondary metabolites, such as phenolic compounds [7], carotenoids [8], vitamins [9, 10] and glucosinolates [11]. On the other hand, other studies have not found the same effect on the final fruit quality [12, 13], emphasising the importance of the environmental parameters (i.e. temperature, irradiation incidence, ripeness, irrigation, etc.) and nutrient dilution (different fruit moisture).

The use of pesticides was reported to affect the secondary metabolism of plants, increasing or decreasing the concentrations of phenolic compounds, depending on the mechanism of action of the pesticide [14]. Several herbicides reduce carbon fixation by plants, decreasing the proportion of carbon available for the synthesis of primary and secondary metabolites. Other herbicides block the shikimate pathway, reducing the synthesis of aromatic amino acids at the onset of the synthesis of phenolic compounds [15].

The quantity and quality of the bioactive compounds in fruit is strongly related to its genotype [16–19]. Plums have a wide genetic basis that is reflected in different contents of antioxidant compounds [20–22]. The nutritional quality of plums also depends on several pre-harvest and post-harvest factors such as irrigation [23], salinity [24], the rootstock used [25], mulching [26] and postharvest treatments [27] such as methyl jasmonate and methyl salicylate [28].

Plum fruits contain important secondary metabolites such as flavonoids and phenolic acids with a high antioxidant capacity [29]. Phenolic compounds are primarily responsible for plums’ antioxidant capacity [30, 31]. The main group of phenolic compounds in plums are hydroxicinnamic acid derivates (chlorogenic acid, neochlorogenic acid and quercetin) [32].

Anthocyanins are abundant in peel [33]; they are responsible for its colour [34] and are affected by factors such as tree position and shading [35]. Carotenoids are natural fat-soluble pigments with an antioxidant capacity [36], the main one in plums being β-carotene, which is mainly found in peel [37].

## Material and Methods

### Plant material, experimental design and treatments

The study was carried out in 2012 and 2013 in two experimental orchards located at IFAPA ‘Las Torres-Tomejil’ (Seville, Spain) (37°30’ 48” N; 5°57’ 46” W). IFAPA is the owner of the fields used for the experiment and they are intended for research purposes. Thirteen different plum cultivars were chosen (Table 1). The experimental design was a total randomized design with three replicates. The fertilisation applied in the organic orchards was animal manure and cover crop, while the fertilizer used in the conventional orchards is detailed in Daza et al. [38]. The plant protection in the conventional orchards followed integrated management guidelines.

**Table 1:** Harvesting date and colours of peel and flesh of plum cultivars

Genotype	Colour^1^		Harvesting date	
	Peel	Flesh	2012	2013
G. Japan	Yellow	Yellow	28/06	25/06
Showtime	Red-Purple	Yellow	19/06	21/06
Sapphire	Purple	Yellow	09/07	25/06
Santa Rosa	Red	Yellow	02/07	26/06
Souvenir	Red-pink	Yellow	09/07	11/07
Black Amber	Dark-purple	Amber	09/07	01/07
Fortune	Red	Amber	26/07	17/07
Friar	Dark-purple	Amber	30/07	31/08
Primetime	Dark-purple	Red	24/07	15/07
F. Larry Ann	Dark Red	Yellow	13/08	05/08
Laetitia	Red	Orange-Yellow	20/08	05/08
Songold	Green-Yellow	Yellow	27/08	06/08
Plumlate	Dark-purple	Amber	17/09	02/09

^1^Daza et al. 2012

### Sample preparation

Eight fruits per tree and six trees per cultivar and treatment were harvested at commercial maturity (Table 1) and transported to the laboratory for analysis. Then, the flesh and pit were separated and each sample frozen in liquid nitrogen and milled. Next, the plum powder was lyophilised and ground again.

### Extraction method for bioactive compounds

Two different approaches were followed for quantifying the bioactive compounds: hydrophilic and lipophilic extractions.

For the hydrophilic extraction, 0.1 g of freeze-dried plum fruits were mixed with 2 ml of acetone:water (50:50 v/v). The mixtures were vortexed for 1 min then sonicated for 15 min and finally centrifuged at 5000 rpmfor 15 min at 4°C. The supernatants were transferred to vials, stored at −80°C, and later used to analyse total phenolics, total anthocyanins, and antioxidant capacity (FRAP and ABTS).

For the lipophilic extraction, 0.1 g of freeze-dried plum fruits were mixed with 2 ml of acetone:hexane (4.6 v/v). The mixtures were vortexed for 2 min and then centrifuged at 5000 rpm for 15 min at 4°C. The supernatants were transferred to vials, stored at −80°C, and later used to analyse carotenoids and antioxidant capacity (ABTS).

### Total anthocyanins

The total anthocyanin content (TAC) was determined following the pH differential method previously described by Giusti and Wrolstad [39]. The extract was diluted in a pH 1.0 solution (25mM KCl) and in a pH 4.5 solution (0.4 M CH_3_CO_2_Na). The absorbance of each well was measured at 535 nm and 700 nm after 15 min at 25°C with a microplate spectrophotometer (Thermo Scientific Multiskan GO). Absorbance readings were converted into total milligrams of cyanidin 3-glucoside per 100 g of dry-weight using the molar extinction coefficient of 23900. The absorbance was calculated using the following equation:

$$\;A=\;{\left(A_{535}-A_{700}\right)}_{pH1.0}–\;{\left(A_{535}-A_{700}\right)}_{pH\;4.5}{}_{}$$

### Total Phenolic compounds

Total polyphenols content (TPC) was determined with the Folin-Ciocalteau reagent using the method of Slinkard and Singleton [40]. First, 10 μl of the hydrophilic extract was mixed with 175 μl of distilled water and subsequently with 12 μl of Folin-Ciolcalteau reagent. After 3 min, 30 μl of a 20% aqueous sodium carbonate solution were added. The samples were left to stand for 1 hour and were then read at 765 nm with a spectrophotometer (previously described) and compared with a known concentration range of similarly-prepared gallic acid standards. The results were expressed as milligrams of gallic acid equivalents per 100 g of fresh-weight (mg GAE/100 g FW).

### Carotenoids

Total carotenoid content (TCC) was determined following the method described by Nagata and Yamashita [41]. Absorbance of the lipophilic extract was measured at 453, 505, 645 and 663 nm. The concentration of carotenoids was calculated with the following equation:

$$ß-carotenoid\;=\;0.216\;A_{663}–\;1.22\;A_{645}–\;0.304\;A_{505}+\;0.452\;A_{453}{}_{}$$

### Antioxidant capacity

The antioxidant capacity of the hydrophilic extract was measured by the ABTS (ABTSh) and FRAP assays. In the case of the lipophilic extract, the antioxidant capacity was measured only by the ABTS assay (ABTS-l).

#### ABTS^•+^ radical scavenging activity

The antioxidant capacity of the samples was also measured by the degree of suppression of the ABTS^•+^ radical cation produced by the reaction of ABTS^•+^ (2,2-azino-di-[3-ethylbenzthiazoline sulphonate]). The data were compared with the antioxidant activity of standard amounts of Trolox.

The stock solution was prepared with 8 ml of water, 1 ml of acetate buffer (0.1 M, pH 5), 1 ml of ABTS (5.5 mg/ml) and a small amount of MnO_2_ (to activate the ABTS radical cation). The solution was filtered through a filter of 0.45 μm to remove the excess MnO_2_. The radical reagent was prepared by diluting the stock solution with pure water to an absorbance equal to 1 measured at 414 nm. Two μl of previously-diluted hydrophilic extractand 200 μl of radical reagent were added to the plate. After shaking in the microplate reader, the absorbance of each well was measured at 414 nm after 50 min at 25°C with the spectrophotometer described above.

To measure the antioxidant capacity in the lipophilic extract, the ABTS radical cation was activated with potassium persulfate and the mixture was left to stand in the dark at room temperature for 12-16 hours before use. The solution was diluted with ethanol to an absorbance of 1 at 414 nm. Next, 40 μl of lipophilic extract was added to 180 μl of ABTS solution. Absorbance was measured at 414 nm after 50 min at 25°C.

#### Ferric reducing antioxidant capacity (FRAP)

The FRAP assay was performed according to the method of Benzie and Strain [42] with some modifications. In short, the FRAP reagent was prepared by mixing 0.3 M acetate buffer (pH 3.6), a solution of 10 mM TPTZ in 40 mM HCl, and 20 mM FeCl_3_6H_2_O at 10:1:1 (v/v/v). Then, 2 μl of the previously diluted sample were added to the well containing 200 μl of FRAP reagent. After shaking in the microplate reader, the absorbance of each well was measured at 593 nm after 40 min at 37°C with the spectrophotometer described above.

The antioxidant capacity (ABTS^•+^ and FRAP assays) was expressed as μmol of trolox equivalent per 100 gram of fresh weight (μmol TE/100 g FW).

### Organic acids (*Ascorbic*, *Citric*, *Fumaric*, *Malic*, *Shikimic*, *Succinic and Tartaricacid*)

Freeze-dried plum (100 mg) was extracted with 3% meta-phosphoric acid in water (v/v) (3 ml). The samples were refrigerated for 10 min and vortexed for 30 seconds every 5 min. The samples were centrifuged at 5000 rpm for 15 min at 4°C and filtered through a 0.45 μm pore-size filter. The extracts were immediately measured by UHPLC-DAD.

The organic acids were measured using a Dionex Ultimate 3000 UHPLC system (Thermo Scientific) equipped with a photodiode array with a multiple wavelength detector. A 4.0 x 250 mm, 5 μm Acclaim organic acid column equipped with an Acclaim organic acid guard column (4.6 x 10 mm, 5 μm) was used. The mobile phase consisted of 0.2% metaphosphoric acid in water adjusted to pH 2.5 using metaphosphoric acid. The flow rate was 0.6 ml min^-1^ (isocratic) and the sample injection volume was 20 μl. The column temperature was set at 35°C. Peaks were recorded at 210 nm for malic, tartaric, citric, fumaric, shikimic and succinic acids and at 260 nm for ascorbic acid. All the above mentioned organic acids were identified and quantified using an external calibration curve prepared with authentic standards and the concentrations expressed as mg/100 g of fresh weight (FW).

The total organic acids content (TOAC) was determined, calculated as the sum of the individual compounds.

### Statistical analysis

The statistical analysis was performed using Statistix software (v. 9.0). Values are given as means. The data were subjected to an analysis of variance (ANOVA), followed by a comparison of means according to the least significant difference (LSD) test. Differences at P<0.05 were considered to be statistically significant.

## Results and Discussion

### Effect of cultivation system

The organic plums had a higher antioxidant capacity (ABTS-h, ABTS-l and FRAP assays), TPC and TAC than the conventional ones (Table 2). The values differed by between 5–10%. TCC was not affected by the method of cultivation.

**Table 2:** Effects of cultivation system, genotype and year of harvest on the anthocyanins, polyphenols, antioxidant capacity, and carotenoids of plums.

	Hydrophilic extraction				Lipophilic extraction	
	TAC (mg GAE/100 g FW)	TPC (mg GAE/100 g FW)	ABTS-h (umols TEAC/100 g FW)	FRAP (umols TEAC/100 g FW)	ABTS-l (umols TEAC/100 g FW)	TCC(mg/100 g FW)
**Cultivation system (CS)**						
Conventional	6.21 b	139.3 b	1158 b	1353 b	5.41 b	0.18
Organic	7.30 a	150.8 a	1253 a	1495 a	5.93 a	0.19
*Significance*	*	***	***	***	**	ns
**Genotype (G)**						
G. Japan	-	71.7 h	552 f	615 h	5.15 de	0.14 de
Showtime	4.25 cd	255.7 a	2651 a	2375 a	5.65 cd	0.28 b
Sapphire	2.10 def	128.1 ef	1057 cd	1125 efg	3.85 f	0.14 de
Santa Rosa	2.72 def	93.2 g	665 f	775 h	3.90 f	0.13 de
Souvenir	0.55 f	118.2 f	943 de	1018 g	6.00 cd	0.26 b
Black Amber	29.82 a	150.2 d	1168 c	2023 b	5.60 cd	0.21 c
Fortune	2.21 def	139.5 de	1164 c	1334 d	7.21 b	0.21 c
Friar	18.17 b	199.4 b	1485 b	2076 b	5.97 cd	0.16 d
Primetime	6.40 c	177.8 c	1489 b	1913 b	4.62 ef	0.07 f
F. Larry Ann	3.12 de	129.4 ef	1144 c	1295 de	6.31bc	0.35 a
Laetitia	0.70 ef	119.2 f	884 e	1089 fg	5.36 de	0.11 e
Songold	-	169.3 c	1370 b	1627 c	8.38 a	0.33 a
Plumlate	4.25 cd	134.2def	1099 c	1247 def	5.76 cd	0.07 f
*Significance*	***	***	***	***	***	***
**Year (Y)**						
2012	4.75 b	133.1 b	1163 b	1265 b	5.44 b	0.22 a
2013	8.76 a	157.0 a	1248 a	1583 a	5.91 a	0.16 b
*Significance*	***	***	***	***	*	***
G*CS	ns	ns	Ns	ns	Ns	ns
G*Y	***	***	***	***	***	***
CS*Y	ns	ns	*	*	Ns	ns
G*Y*CS	ns	ns	Ns	ns	*	ns

TAC = Total anthocyanins content, TPC = total polyphenolic content, ABTS-h = ABTS antioxidant capacity assay on hydrophilic extraction, FRAP = FRAP antioxidant capacity assay on hydrophilic extraction, ABTS-l = ABTS antioxidant assay in lipophilic extraction and TCC = total carotenoid content. Means with different letters in the same column present significant differences. Significance level: ns = non significant,

* = *P* <0.05,

** = *P* <0.01,

*** = *P* <0.001

Several publications have shown that lower nitrogen application results in a higher accumulation of phenolic compounds, whereas higher levels of nitrogen fertilization promote the formation of carotenoids and chlorophylls [43]. The first is in agreement with our data (TAC, TPC), while the latter would suggest a higher TCC value in conventional plums. However, other authors [44] observed that the TCC is highly temperature dependent and this could be the reason for the results obtained.

Organic growing systems are related to a certain level of stress (restricted and limited use of pesticides and fertilizers), which could lead to the accumulation of secondary metabolites responsible for plant defence [7, 45]. The most important secondary metabolites in plums are polyphenols [32], whose content commonly increases in stressful conditions, as was observed in the samples analyzed. Shikimic acid is the precursor of phenylalanine [46], and therefore precursor of the phenylpropanoid biosynthesis [47]. In our case, a higher amount of shikimic acid and TPC were found in the organic orchards. These results were in agreement with the literature published in Prunus genus [8] and other fruits [48, 49, 50, 51].

Organic acids have an important role in cell metabolism and also contribute to the final quality of fresh fruit. Seven organic acids were identified and quantified: malic, citric, tartaric, fumaric, succinic, ascorbic and shikimic acids. We found many publications about ascorbic acid and plums [37, 52, 53, 54] and prunes [55] while deeper studies regarding overall study of organic acids in *Prunus salicina* L. [56] were scarce.

In our case, the plums cultivated in an organic system obtained higher values for TOAC (1475 mg/100g FW vs1409 mg/100g FW for conventional ones), therefore, the production system was shown to have a significant effect (Table 3). Additionally, the plums grown under organic conditions showed significantly higher concentrations of malic, succinic, tartaric and shikimic acids. Other organic acids, such as citric, ascorbic and fumaric acid, were not affected by the cultivation system. Regarding this results, Lombardi et al [15] did not find differences in citric and malic acid content on *Prunus domestica* L. between organic and conventional orchards. These results are partly in agreement with the data we obtained. This fact could be related with the difference between the two species under study (*Prunus salicina* L. and *Prunus domestica* L.).

**Table 3:** Effects of cultivation system, genotype and year of harvest on the malic, citric, ascorbic, succinic, tartaric, fumaric, shikimic and total organic acids content in plums.

	Ascorbic acid	Citric acid	Fumaric acid	Malic acid	Shikimic acid	Succinic acid	Tartaric acid	Total organic acids
**Cultivation system (CS)**								
Conventional	1.62	24.12	0.49	1313 b	3.65 b	5.84 b	60.18 b	1409 b
Organic	1.66	24.00	0.50	1369 a	3.90 a	7.01 a	67.67 a	1475 a
*Significance*	ns	ns	ns	**	***	*	***	***
**Genotype (G)**								
Golden Japan	1.48 de	22.80 cde	0.27 f	1662 c	2.68 e	15.21 a	48.68 efgh	1754c
Showtime	2.02 bc	38.16 a	0.68 c	2169 a	3.30 d	4.84 def	85.30 c	2304 a
Sapphire	2.49 a	20.67 e	1.24 a	1224 efg	3.27 d	2.33 f	32.02 i	1286 fg
Santa Rosa	1.47 de	23.22 cd	0.38 de	1804 b	3.33 d	2.85 f	41.45 h	1877 b
Souvenir	1.26 ef	23.74 c	0.27 f	1469 d	3.71 c	7.92 bc	55.34 e	1562 d
Black amber	2.29 ab	21.33 de	0.28 ef	1179 fg	4.75 a	8.50 b	53.51 ef	1270 g
Fortune	1.79 c	22.67 cde	0.62 c	1280 e	3.85 bc	4.14 def	97.15 b	1410 e
Friar	2.13 b	28.34 b	0.83 b	1216 efg	4.92 a	6.70 bcd	107.2 a	1367 ef
Primetime	1.73 cd	21.93 cde	0.38 de	1236 ef	4.12 b	5.83 cde	46.50 fgh	1316 fg
F. Larry Ann	1.50 de	23.79 c	0.40 d	1181fg	4.97 a	4.55 def	72.58 d	1289 fg
Laetitia	0.73 g	13.19 f	0.31 def	1055 h	2.84 e	3.32 ef	45.53 gh	1120 h
Songold	1.06 f	29.02 b	0.37 def	1151g	3.39 d	9.00 b	50.42 efg	1245 g
Plumlate	1.38 e	23.90 c	0.36 def	810 i	3.95 bc	8.30 bcd	95.36 b	943 i
*Significance*	***	**	***	***	***	***	***	***
**Year (Y)**								
2012	1.75 a	24.89 a	0.57 a	1233 b	3.88 a	5.05 b	54.61 b	1324 b
2013	1.53 b	23.22 b	0.42 b	1450a	3.67 b	7.80 a	73.23 a	1560 a
*Significance*	***	***	***	***	***	***	***	***
**G x CS**	ns	ns	ns	*	ns	ns	ns	*
**G x Y**	***	***	***	***	***	***	***	***
**CS x Y**	ns	***	ns	ns	ns	ns	ns	ns
**G x Y x CS**	ns	**	ns	**	ns	ns	*	**

Unit = mg/100 g fresh weight. Means with different letters in the same column present significant differences. Significance level:ns = non significant,

* = *P* <0.05,

** = *P* <0.01,

*** = *P* <0.001

Stressful conditions such as water supply [57], soil conditions [58], etc., could lead to a higher amount of organic acids in plums, depending on tree responses. Several authors [59] suggests that higher amounts of nitrogen fertilization lead to a reduction in ascorbic acid concentration in many fruits [60, 61] and vegetables [62, 63], which is in contrast with the results obtained in our research where no differences were found between the organic and conventional cultivation methods, in which fertilization was very different. This controversial matter is supported by other publications in which similar results to ours were found [62, 64, 65, 66]

### Effect of genotype

Significant differences in the phytochemical content were found among different plum genotypes and the biosynthesis of phytochemical compounds in plants is known to be strongly influenced by genotype [67]. 'Showtime' showed the highest values for TPC, ABTS-h, FRAP among all the cultivars as well as a high TCC. 'Songold' recorded the highest values for carotenoids and ABTS-l. 'Golden Japan' and 'Santa Rosa' presented the lowest values for ABTS-h and FRAP. 'Golden Japan' also had the lowest TPC. 'Black amber' was the cultivar with the highest anthocyanin content, followed by 'Friar', both of these having dark purple skin. The quantification of TAC was feasible for all the cultivars studied except for 'Songold' and 'Golden Japan', cultivars with yellow skin and flesh.

'Golden Japan' was previously characterized as a cultivar with a low antioxidant capacity [68], in agreement with our results; while 'Showtime' presented the highest (2650.6 μmol ET/100 g FW) antioxidant capacity. These results showed an important genotype effect in the sample of plums under study.

There were significant differences among cultivars in TOAC (Table 3). The highest values were measured for the ‘Showtime’ cultivar (2304 mg/100g FW) followed by 'Santa Rosa' (1877 mg/100g FW), and the lowest ones were found for 'Plumlate' (943 mg/100g FW).

Malic acid was the major contributor to the TOAC with a range between 86–96% depending on the cultivar. These findings were in agreement with Singh et al. [56]. Tartaric acid was the second most abundant organic acid followed by citric, succinic, shikimic, ascorbic and fumaric acids.

‘Showtime’ was the cultivar with the highest content of malic and citric acids, both related to sour taste. The highest concentrations for ascorbic acid were found for the 'Sapphire' and 'Black amber' cultivars.

The TOAC data found in our study were consistent with the literature [56] and differences in the TOAC between cultivars were also previously described [69]. Ascorbic acid content in plums is low [37, 70] so its contribution to their antioxidant capacity is practically negligible.

### Year effect

The plums harvested in 2013 showed a significantly higher TPC, TAC, and antioxidant capacity (ABTS-h, FRAP and ABTS-l), while those harvested in 2012 showed significant higher TCC values (Table 2). Regarding organic acids, the plums harvested in 2013 showed higher values for TOAC, malic, succinic and tartaric acids, while higher values for citric, ascorbic, fumaric and shikimic acids were found for the samples harvested in 2012 (Table 3).

Many parameters are involved in the factor ‘year’, such as plant nutrient imbalances, irrigation, crop load, fruit canopy position and environmental parameters [71].

Climate data were studied to identify the influence of environmental conditions on phytochemical compounds during the two years under study. The main difference observed between the two years was the value for the total radiation (TR). In 2012, TR was between 3 and 8% higher (mean monthly values) than in 2013.

The mechanism of carotenoid biosynthesis has been shown to be light-dependent [72], which may explain our results, which show greater carotenoid biosynthesis in 2012 (Table 2).

Other studies involving plums (‘Laetitia’ and ‘Songold’) found that fruit grown under reduced light conditions were consistently less mature than those exposed to full sunlight [73]. In our study, a higher content of organic acids was found in 2013, the year with a lower total radiation. Thus, the lower radiation found in 2013 could have produced less mature plums and therefore a higher content of organic acids.

### Interaction effects

All the cultivars studied had higher bioactive compounds content in organic cultivation system while ‘cultivation system and genotype’ interaction was not significant for TAC, TPC, ABTS-l and TCC (Table 2). This means that cultivation system affected above mentioned measurements in the same way for all studied cultivars.

The 'genotype x year' interaction was significant and therefore, the genotype responses to the environmental factors (year effect) were different. ‘Golden Japan’ showed different concentrations of all the parameters studied in both years. Therefore, the bioactive compounds of the ‘Golden Japan’ cultivar were mainly affected by environmental conditions. Hydrophilic bioactive compounds for ‘Fortune’, ‘Plumlate’, ‘Sapphire’ and ‘Santa Rosa’ were not significantly different in both years and ‘Black Amber’ demonstrated the same behaviour for the lipophilic extract and carotenoid content (S1 Table).

The 'cultivation system x year' interaction was significant for the FRAP assay and ABTS-h. In this case, the plums harvested in 2013 showed no significant differences between both growing systems, but the organic plums in 2012 showed a higher antioxidant capacity than the conventional ones (S1 Table).

### Correlations

High correlations (S2 Table) between TPC and both antioxidant assays were observed (r^2^ ≈ 0.89 for both). Similar results were previously reported in plums [32, 37, 74].

No correlation between TPC and TAC (R^2^ = 0,401) was found. Previous studies observed that TAC and TPC were not well correlated in other fruits with high TAC and TPC values [75].

Moreover, the ascorbic acid content did not correlate well with different antioxidant capacity assays (S2 Table), so polyphenols compounds (TPC) make the biggest contribution to the antioxidant capacity of plums.

The results of the FRAP and ABTS-h assays show a linear correlation (R^2^ = 0.794, *P*<0.001, N = 156), so both methods may be used indistinctly to evaluate the antioxidant activity in these type of fruits.

## Conclusions

Organic plum samples were found to have higher values of TAC, TPC and antioxidant capacity than conventional ones while no differences in TCC were found. The main bioactive compounds contributing to the total antioxidant capacity of plums are phenols. Genotype significantly affected the content of different bioactive compounds. ‘Showtime’ had the highest TPC values and ‘Golden Japan’ and ‘Santa Rosa’ the lowest.

In conclusion, the organic growing system produced plums with a higher content of bioactive compounds, in addition to a lower impact on the environment.

## Supporting Information

S1 Table ANOVA interactions between cultivation system, genotype and year factors, and effects on total anthocyanins, polyphenols, antioxidant capacity and carotenoids values. Means with different letters in the same column show significant differences. P<0.05. TAC = Total anthocyanins content. TPC = Total polyphenols content. ABTS-h = ABTS hydrophilic extract. TCC = Total carotenoids content. ABTS-l = ABTS lipophilic extract. CS: Cultivation system. G: genotype. Y: year.
(DOCX)

S2 Table Pearson's correlation coefficients of hydrophilic extraction assays (TAC,TPC, ABTS-h, FRAP), lipophilic extraction assays (ABTS-l and TCC) and organic acids values. ^ns^ = non significant, * = significant *P* <0.05, ** = significant *P* <0.01, *** = significant *P* <0.001.
(DOCX)
