# Supplementary material for: Effect of Organic and Conventional Management on Bio-Functional Quality of Thirteen Plum Cultivars (Prunus salicina Lindl.)
Source: PLoS One. 2015 Aug 27;10(8):e0136596. doi: 10.1371/journal.pone.0136596 (PMC4551474; doi:10.1371/journal.pone.0136596)
Supplement: S1 Table — Means with different letters in the same column show significant differences. P<0.05. TAC = Total anthocyanins content. TPC = Total polyphenols content. ABTS-h = ABTS hydrophilic extract. TCC = Total carotenoids content. ABTS-l = ABTS lipophilic extract. CS: Cultivation system. G: genotype. Y: year. (DOCX) [file pone.0136596.s001.docx]

| **Supporting 1 Table. ANOVA interactions between cultivation system, genotype and year factors and effects in total anthocyanins, polyphenols, antioxidant capacity and carotenoids values** | | | | | | |
| --- | --- | --- | --- | --- | --- | --- |
|  | **TAC**  **(mg/100 g FW)** | **TPC**  **(mg GAE/100 g FW)** | **ABTS-h**  **(umols ET/100 g FW)** | **FRAP**  **(umols ET/100 g FW)** | **ABTS-l**  **(umols ET/100 g FW)** | **TCC**  **(mg/100 g FW)** |
| **CS x G** |  |  |  |  |  |  |
| Golden Japan conv | - | 64.86 | 514.7 | 569.4 | 4.7571 | 0.1283 |
| Golden Japan org | - | 78.43 | 588.3 | 660.0 | 5.5437 | 0.1467 |
| Showtime conv | 4.361 | 259.95 | 2622.4 | 2285.7 | 4.8001 | 0.3067 |
| Showtime org | 4.147 | 251.53 | 2678.8 | 2464.5 | 6.4906 | 0.2517 |
| Sapphire conv | 1.832 | 126.48 | 1082.8 | 1113.9 | 3.7575 | 0.1350 |
| Sapphire org | 2.372 | 129.78 | 1031.0 | 1136.7 | 3.9347 | 0.1367 |
| Santa Rosa conv | 2.223 | 78.34 | 529.3 | 622.4 | 4.0004 | 0.1133 |
| Santa Rosa org | 3.219 | 108.1 | 801.2 | 927.8 | 3.8013 | 0.1450 |
| Souvenir conv | 0.560 | 116.80 | 928.3 | 1032.4 | 5.7148 | 0.2283 |
| Souvenir org | 0.542 | 119.59 | 957.9 | 1003.8 | 6.2817 | 0.2850 |
| Black amber conv | 26.872 | 146.32 | 1174.4 | 1968.4 | 5.4585 | 0.2100 |
| Black amber org | 32.768 | 154.17 | 1160.5 | 2076.7 | 5.7361 | 0.2133 |
| Fortune conv | 2.075 | 135.92 | 1094.1 | 1232.7 | 7.0209 | 0.2133 |
| Fortune org | 2.348 | 143.1 | 1234.1 | 1435.3 | 7.3980 | 0.2083 |
| Friar conv | 17.298 | 180.44 | 1452.8 | 1958.7 | 5.4750 | 0.1600 |
| Friar org | 19.033 | 218.41 | 1517.7 | 2193.1 | 6.4641 | 0.1533 |
| Primetime conv | 6.222 | 169.84 | 1372.1 | 1855.2 | 4.1191 | 0.0750 |
| Primetime org | 6.582 | 185.66 | 1606.4 | 1970.9 | 5.1266 | 0.0667 |
| Larry Ann conv | 2.970 | 126.13 | 1159.1 | 1325.5 | 6.2884 | 0.3267 |
| Larry Ann org | 3.275 | 132.68 | 1129.7 | 1263.7 | 6.3312 | 0.3717 |
| Laetita conv | 0.708 | 116.16 | 842.4 | 1067.2 | 5.2957 | 0.1117 |
| Laetita org | 0.697 | 122.19 | 925.8 | 1110.9 | 5.4167 | 0.1100 |
| Songold conv | - | 165.80 | 1305.3 | 1500.0 | 8.7518 | 0.3067 |
| Songold org | - | 172.84 | 1434.3 | 1753.4 | 7.9993 | 0.3567 |
| Plumlate conv | 3.188 | 123.77 | 973.4 | 1061.5 | 4.8969 | 0.0733 |
| Plumlate org | 5.307 | 144.64 | 1224.1 | 1433.3 | 6.6218 | 0.0717 |
| **G x Y** |  |  |  |  |  |  |
| Golden Japan 2012 | - | 95.63 l | 784.9 | 866.0 lmn | 6.3024 cdefg | 0.2067 fg |
| Golden Japan 2013 | - | 47.67 m | 318.1 | 363.4 o | 3.9984 kl | 0.0683 m |
| Showtime 2012 | 2.844 efgh | 235.08 b | 2499.1 | 2070.4 cd | 5.1575 ghijk | 0.3300 b |
| Showtime 2013 | 5.663 def | 276.40 a | 2802.1 | 2679.9 a | 6.1332 cdefgh | 0.2283 def |
| Sapphire 2012 | 2.523 fgh | 126.77 hijk | 1066.1 | 1149.5 ij | 3.7921 kl | 0.1750 ghi |
| Sapphire 2013 | 1.680 gh | 129.49 hijk | 1047.7 | 1101.2 ijk | 3.9001 kl | 0.0967 klm |
| Santa Rosa 2012 | 3.958 efgh | 93.89 l | 730.1 | 868.5 klmn | 4.4528 jkl | 0.1683 ghij |
| Santa Rosa 2013 | 1.485 h | 92.46 l | 600.5 | 681.8 n | 3.3488 l | 0.0900 lm |
| Souvenir 2012 | 0.570 h | 99.55 l | 778.4 | 816.4 mn | 4.8094 ijk | 0.2567 cde |
| Souvenir 2013 | 0.532 h | 136.85 gh | 1107.8 | 1219.8 ij | 7.1871 bcd | 0.2567 cde |
| Black amber 2012 | 15.593 c | 108.31 jkl | 985.2 | 1327.1 ghi | 4.9906 hijk | 0.2017 fg |
| Black amber 2013 | 44.047 a | 192.18 de | 1349.7 | 2718.0 a | 6.2040 cdefgh | 0.2217 efg |
| Fortune 2012 | 3.342 efgh | 139.30 gh | 1176.9 | 1344.1 ghi | 7.0044 bcde | 0.2750 bcd |
| Fortune 2013 | 1.082 h | 139.63 gh | 1151.3 | 1323.8 ghi | 7.4144 abc | 0.1467 hijk |
| Friar 2012 | 8.678 d | 174.41 ef | 1505.3 | 1794.3 ef | 4.6188 jkl | 0.1217 jkl |
| Friar 2013 | 27.653 b | 224.44 bc | 1465.1 | 2367.5 b | 7.3203 abcd | 0.1917 fgh |
| Primetime 2012 | 6.493 de | 147.47 gh | 1404.6 | 1568.2 fg | 3.8968 kl | 0.0850 lm |
| Primetime 2013 | 6.310 de | 208.03 cd | 1573.9 | 2257.8 bc | 5.3488 fghij | 0.0567 m |
| Larry Ann 2012 | 2.435 fgh | 109.54 ijkl | 1012.7 | 1084.5 ijkl | 5.9987 defghi | 0.3967 a |
| Larry Ann 2013 | 3.810 efgh | 149.26 gh | 1465.1 | 1504.7 gh | 6.6209 cdef | 0.3017 bc |
| Laetita 2012 | 0.625 h | 107.42 kl | 822.1 | 967.3 jklm | 4.9678 hijk | 0.1233 ijkl |
| Laetita 2013 | 0.780 h | 130.93 hij | 946.1 | 1210.7 ij | 5.7446 efghij | 0.0983 klm |
| Songold 2012 | - | 157.57 fg | 1282.9 | 1342.4 ghi | 8.5517 a | 0.4333 a |
| Songold 2013 | - | 181.08 e | 1456.7 | 1911.0 de | 8.1994 ab | 0.2300 def |
| Plumlate 2012 | 5.192 defg | 135.92 gh | 1065.8 | 1261.5 hi | 6.1438 cdefgh | 0.0783 lm |
| Plumlate 2013 | 3.303 efgh | 132.50 hi | 1131.7 | 1233.2 i | 5.3750 fghij | 0.0667 m |
| **CS x Y** |  |  |  |  |  |  |
| conventional 2012 | 4.2435 | 126.04 | 1089.6 b | 1160.1 c | 5.0353 | 0.2156 |
| conventional 2013 | 8.1764 | 152.55 | 1226.0 a | 1546.5 a | 5.7857 | 0.1518 |
| organic 2012 | 4.2435 | 140.25 | 1235.7 a | 1370.7 b | 5.8396 | 0.2231 |
| organic 2013 | 9.3409 | 161.44 | 1270.4 a | 1618.5 a | 6.0289 | 0.1641 |
| Means with different letters in the same column present significant differences. P<0.05. TAC = Total anthocyanins content. TPC = Total polyphenols content. ABTS-h = ABTS hydrophilic extract. TCC = Total carotenoids content. ABTS-l = ABTS lipophilic extract. CS: Cultivation system. G: genotype. Y: year. | | | | | | |
