# Supplementary material for: Effect of Organic and Conventional Management on Bio-Functional Quality of Thirteen Plum Cultivars (Prunus salicina Lindl.)
Source: PLoS One. 2015 Aug 27;10(8):e0136596. doi: 10.1371/journal.pone.0136596 (PMC4551474; doi:10.1371/journal.pone.0136596)
Supplement: S2 Table — ns = non significant, * = significant P <0.05, ** = significant P <0.01, *** = significant P <0.001. (DOCX) [file pone.0136596.s002.docx]

| **Supporting 2 Table. Pearson's correlation coefficients of hidrophilic extraction assays (TAC,TPC, ABTS-h, FRAP), lipophilic extraction assays (ABTS-l and TCC) and organic acids values** | | | | | | | | | | | | | | |
| --- | --- | --- | --- | --- | --- | --- | --- | --- | --- | --- | --- | --- | --- | --- |
|  | Hidrophilic extraction | | | |  | Lipophilic extraction | |  | Organic acids | | | | | |
|  | TAC | TPC | ABTS-H assay | FRAP assay |  | ABTS-L assay | TCC |  | Ascorbic acid | Citric acid | Fumaric acid | Malic acid | Shikimic acid | Succinic acid |
| TPC | 0.395^***^ |  |  |  |  |  |  |  |  |  |  |  |  |  |
|  |  |  |  |  |  |  |  |  |  |  |  |  |  |  |
| ABTS-H assay | 0.180^*^ | 0.886^***^ |  |  |  |  |  |  |  |  |  |  |  |  |
|  |  |  |  |  |  |  |  |  |  |  |  |  |  |  |
| FRAP | 0.647^***^ | 0.891^***^ | 0.794^***^ |  |  |  |  |  |  |  |  |  |  |  |
|  |  |  |  |  |  |  |  |  |  |  |  |  |  |  |
| ABTS-L assay | 0.189^*^ | 0.431^***^ | 0.351^***^ | 0.402^***^ |  |  |  |  |  |  |  |  |  |  |
|  |  |  |  |  |  |  |  |  |  |  |  |  |  |  |
| TCC | 0.077^ns^ | 0.128^ns^ | 0.208^*^ | 0.119^ns^ |  | 0.443^***^ |  |  |  |  |  |  |  |  |
|  |  |  |  |  |  |  |  |  |  |  |  |  |  |  |
| Ascorbic acid | 0.262^**^ | 0.240^**^ | 0.305^***^ | 0.269^**^ |  | -0.1312^ns^ | 0.102^ns^ |  |  |  |  |  |  |  |
|  |  |  |  |  |  |  |  |  |  |  |  |  |  |  |
| Citric acid | 0.084^ns^ | 0.645^***^ | 0.716^***^ | 0.500^***^ |  | 0.303^***^ | 0.308^***^ |  | 0.337^***^ |  |  |  |  |  |
|  |  |  |  |  |  |  |  |  |  |  |  |  |  |  |
| Fumaric acid | -0.018^ns^ | 0.156^ns^ | 0.178^*^ | 0.085^ns^ |  | -0.053^ns^ | 0.112^ns^ |  | 0.467^***^ | 0.194^*^ |  |  |  |  |
|  |  |  |  |  |  |  |  |  |  |  |  |  |  |  |
| Malic acid | -0.101^ns^ | 0.409^***^ | 0.487^***^ | 0.260^**^ |  | 0.041^ns^ | 0.195^*^ |  | 0.137^ns^ | 0.596^***^ | -0.007^ns^ |  |  |  |
|  |  |  |  |  |  |  |  |  |  |  |  |  |  |  |
| Shikimic acid | 0.246^**^ | 0.104^ns^ | 0.082^ns^ | 0.176^*^ |  | 0.148^ns^ | 0.254^**^ |  | 0.401^***^ | 0.267^**^ | -0.083^ns^ | -0.154^ns^ |  |  |
|  |  |  |  |  |  |  |  |  |  |  |  |  |  |  |
| Succinic acid | 0.468^***^ | 0.366^***^ | 0.177^*^ | 0.379^***^ |  | 0.334^***^ | 0.075^ns^ |  | 0.015^ns^ | 0.131^ns^ | -0.205^*^ | -0.007^ns^ | 0.130^ns^ |  |
|  |  |  |  |  |  |  |  |  |  |  |  |  |  |  |
| Tartaric acid | 0.254^**^ | 0.525^***^ | 0.396^***^ | 0.450^***^ |  | 0.561^***^ | 0.124^ns^ |  | -0.036^ns^ | 0.454^***^ | 0.024^ns^ | 0.053^ns^ | 0.266^**^ | 0.328^***^ |
| ^ns^= non significant, ^*^ = significant *P* <0.05, *^*^ = significant *P* <0.01, ^***^ = significant *P* <0.001 | | | | | | | | | | | | | | |
